# mGWAS-Explorer: Linking SNPs, Genes, Metabolites, and Diseases for Functional Insights

**DOI:** 10.3390/metabo12060526

**Published:** 2022-06-07

**Authors:** Le Chang, Guangyan Zhou, Huiting Ou, Jianguo Xia

**Affiliations:** 1 Department of Human Genetics, McGill University, Montreal, QC H3A 0C7, Canada; le.chang@mail.mcgill.ca (L.C.); huiting.ou@mail.mcgill.ca (H.O.); 2 Institute of Parasitology, McGill University, Montreal, QC H9X 3V9, Canada; guangyan.zhou@mail.mcgill.ca

**Keywords:** mGWAS, SNP, mQTL, metabolomics, pleiotropy, cross-phenotype association analysis, network

## Abstract

Tens of thousands of single-nucleotide polymorphisms (SNPs) have been identified to be significantly associated with metabolite abundance in over 65 genome-wide association studies with metabolomics (mGWAS) to date. Obtaining mechanistic or functional insights from these associations for translational applications has become a key research area in the mGWAS community. Here, we introduce mGWAS-Explorer, a user-friendly web-based platform to help connect SNPs, metabolites, genes, and their known disease associations via powerful network visual analytics. The application of the mGWAS-Explorer was demonstrated using a COVID-19 and a type 2 diabetes case studies.

## 1. Introduction

Genome-wide association studies (GWAS) have identified hundreds of thousands of genetic loci associated with complex diseases. These associations have improved our understanding of the genetic architecture of human diseases [1]. However, translations of these associations into biomedical or pharmaceutical applications have been limited, as the majority of the disease-associated loci reside in the non-coding regions of the genome with no obvious gene targets [2]. Technology advancements in mass spectrometry (MS) and nuclear magnetic resonance (NMR) spectroscopy have allowed GWAS to be carried out with metabolomics (mGWAS) to study genetically influenced metabotypes (GIMs) [3,4]. mGWAS have been very successful in identifying metabolite quantitative trait loci (mQTLs). An mQTL is a locus that is associated with variations in metabolite abundance [3]. In addition to having larger effects compared to loci identified in GWAS of clinical phenotypes in general, many mQTLs can map to genes encoding enzymes or transporters, providing biochemical context for these variations [3,5]. Leveraging these mQTLs to improve our knowledge of metabolism and metabolic disorders for translational applications has become a key research area in the mGWAS community.

mQTLs are characterized by polygenicity and pleiotropy [6,7]. Polygenicity means a single trait is influenced by multiple genes, whereas pleiotropy refers to the phenomenon in which genetic variants affect multiple traits or diseases [8,9]. For instance, one single-nucleotide polymorphism (SNP) can directly affect multiple traits, or different SNPs in high linkage disequilibrium (LD) may exist for more than one trait. Pleiotropy may provide insights into the cause of trait comorbidity and help determine the direction of causal relationships by pointing to shared genetic mechanisms [8,10]. Various strategies have been developed to examine genetic relationships between multiple phenotypes [11,12,13,14,15,16,17,18]. For example, LD score regression is a popular method to assess the genetic correlations of pairwise traits using GWAS summary statistics [13]. Colocalization is another strategy aiming to identify causal variants at two overlapping association signals [14]. These methods have successfully identified pleiotropic genomic regions and addressed fundamental research questions regarding the polygenicity of traits, but it is challenging to scale up these methods to study hundreds of traits at once.

Comprehensive annotations are necessary in order to gain functional insights into SNP–metabolite associations. Many resources are available to support SNP to gene annotation, such as VEP and SNiPA [19,20]. For metabolite annotation, there is a wealth of biochemical knowledge on enzymatic reactions as well as transporters and their substrates. In addition, mapping GWAS results to the protein–protein interaction (PPI) network can potentially augment the association signals [21].

Recently, cross-phenotype association analysis has gained increasing attention [22,23,24,25,26]. It takes a specific SNP and searches for associations across a range of molecular or disease phenotypes, which allows for elucidations of complex networks between phenotypes and their genetic loci. A variety of databases currently exist to store the genotype–phenotype association datasets, including GWAS Catalog [27], PhenoScanner [28], OpenGWAS [29], Open Targets Genetics [30], PheLiGe [31], DisGeNET [32], as well as specific tools for mGWAS, such as the metabolomics GWAS server [33,34]. Valuable tools currently exist to allow users to perform cross-phenotype analysis [35,36,37,38,39,40]. However, these tools do not offer extra support beyond displaying and visualization, and none of them are dedicated to mGWAS. 

There is a clear demand for dedicated bioinformatics resources to support mGWAS data analysis and interpretation. Our overall assumption is that by developing a centralized place for mGWAS datasets and performing deep annotation of the underlying SNPs and metabolites, users can gain valuable functional insights into the statistical associations identified from mGWAS results. 

A network is a valuable approach to depict mGWAS results and allows the dissection of polygenicity and pleiotropy. Heterogeneous networks comprising various types of nodes (e.g., SNPs, genes, metabolites, and diseases) and edges (e.g., statistical or biochemical associations) have been remarkably useful in depicting the complex interplay across biological entities [41]. These network-based approaches have the potential to identify and prioritize therapeutic candidates to generate new hypotheses [42].

Here, we introduce mGWAS-Explorer (https://www.mgwas.ca (accessed on 1 May 2022), a user-friendly web-based platform for network-based integrative analysis and visual exploration of SNPs, genes, metabolites, and diseases. Its key features include:

- Comprehensive collection and deep annotation of SNP–metabolite associations based on data from the 65 mGWAS to date.
- Support for SNP-based, gene-based, and metabolite-based network generation to facilitate interpreting results.
- Powerful network visual analytics system facilitating interactive exploration and built-in topological and functional enrichment analysis.

mGWAS-Explorer also includes a comprehensive list of frequently asked questions (FAQs) and detailed tutorials. Together, these features comprise a powerful platform for functional interpretation and cross-phenotype association analysis of mGWAS datasets.

## 2. Results

### 2.1. Overview of the Curated mGWAS Datasets

Since the first study in 2008 [5], mGWAS with increasing sample sizes and various populations have been conducted, resulting in a continued increase in SNP–metabolite associations. We systematically curated the public mGWAS datasets to date. A summary table of these mGWAS datasets can be found in Table 1. Please note the *p*-value cutoffs are based on significance thresholds of the original studies, as the *p*-values and effect sizes of SNP-metabolite associations may differ across different studies due to the differences in sample sizes, population types, or the metabolomics platforms [4,7]. 

### 2.2. Overview of the mGWAS-Explorer

**Figure 1:**
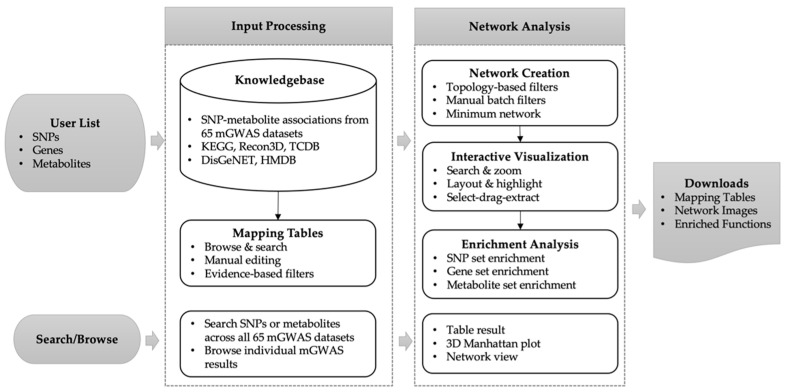
Overview of mGWAS-Explorer workflow. Users can upload different data types. The input will be mapped to the underlying knowledgebases to create mapping tables and networks. The visualization page allows users to intuitively explore the networks to identify important associations as well as to perform topology or functional analysis.

The main workflow of mGWAS-Explorer is summarized in Figure 1. There are three major steps—data input, network creation, and network visual analytics. To begin, users can enter through one of the five modules based on input type. The ‘SNP’ module allows users to explore SNP–gene, SNP–metabolite, or SNP–disease networks. We provide support for LD proxy search to maximize the search by looking for SNPs in LDs with the input SNPs. After SNP to gene mapping, users can choose to include PPIs in the networks. The ‘Gene’ module maps genes to SNPs that are significant in the mGWAS datasets, or to metabolites (i.e., through encoding enzymes or transporters), or known associated diseases. The ‘Metabolite’ module maps metabolites to associated SNPs, genes, or diseases. The ‘Search’ module allows users to search known SNP–gene associations in mGWAS datasets, while the ‘Browse’ module allows users to browse individual mGWAS data in a 3D Manhattan plot or a network view. To start the analysis, users must click a circular button from the mGWAS-Explorer homepage to enter the corresponding data upload page. Various functions are available to allow users to refine the networks. In the last step, the results are shown as interactive networks for visual exploration. Users can easily search, explore, highlight, or perform functional enrichment analysis on the nodes of interest. For instance, double-clicking an edge will display the evidence supporting the relationships. The network results can be downloaded in PNG, SVG format, or as graph files.

### 2.3. Analysis Workflow

**Figure 2:**
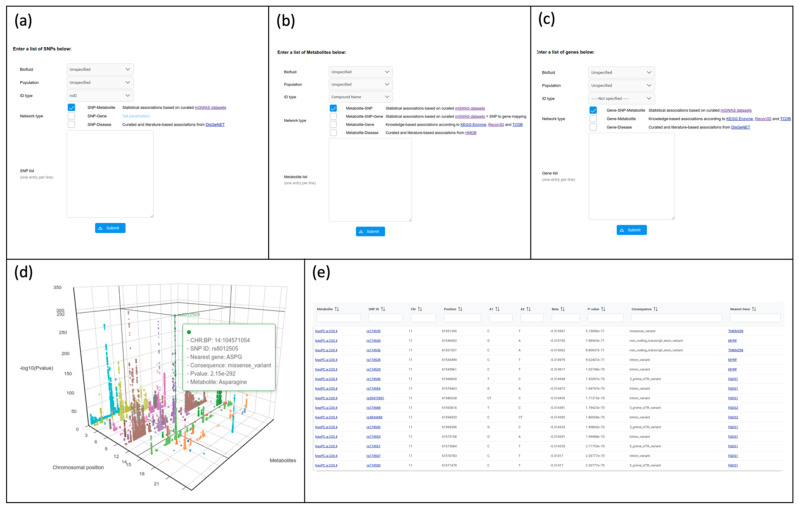
Screenshots of upload pages for SNP (**a**), metabolite (**b**), and gene (**c**) modules. (**d**) A screenshot of a 3D Manhattan plot from the ‘Browse’ module based on the data from Lotta et al. [7]. The x-axis is the position of the SNPs, and the y-axis represents different metabolites, while the z-axis corresponds to the significance of the association; (**e**) a screenshot showing the table view in the ‘Browse’ module.

There are five modules in mGWAS-Explorer corresponding to the five different types of input. Users can upload a list of SNPs, metabolites, or genes (Figure 2a–c); browse individual mGWAS dataset in a 3D Manhattan plot (Figure 2d); or search significant SNP-metabolite associations across all mGWAS datasets (Figure 2e).

#### 2.3.1. Search and Browse

The ‘Search’ module supports searching the association results of the curated 65 mGWAS publications. Meanwhile, the ‘Browse’ module allows users to visually explore the data in a 3D Manhattan plot or network view. The 3D Manhattan plot strengthens the exploration of metabolome-wide pleiotropy at the genome-wide level [39]. Users can mouse-over a dot to see the SNP annotation, including the rsID, CHR:BP, nearest gene, most severe consequence, *p*-value, and metabolite name (Figure 2d).

#### 2.3.2. From SNPs to Networks

Network-based approaches have become increasingly applied to identify shared genetic underpinnings (i.e., pleiotropy) in GWAS, where nodes are SNPs or phenotypes (e.g., metabolites or diseases) and edges represent significant associations [11]. The ‘SNPs’ module supports SNP–metabolite (or metabolite ratios), and SNP–disease network analysis. Optionally, LD proxy search (i.e., population type and r^2^) could be performed to maximize the results. Users also have the flexibility to include PPI networks, as well as to filter on biofluid or population types (Figure 2a).

#### 2.3.3. From Metabolites to Networks

For many GIMs, metabolites can be functionally connected to enzymes, or transporters [3]. The ‘Metabolites’ module allows users to perform either statistical-based or knowledge-based metabolite-gene associations as well as metabolite-disease associations. Users can upload a list of metabolites from the upload page (Figure 2b). mGWAS-Explorer currently accepts either HMDB ID, KEGG ID or compound name. The uploaded list is then mapped to genes, SNPs, or diseases for network creation and subsequent visualization.

#### 2.3.4. From Genes to Networks

The nearest gene mapping approach is suggested to be an effective indicator of true positive genes for mQTLs [44]. In mGWAS-Explorer, users can upload their gene lists in the ‘Genes’ module, the reversed nearest-gene mapping will be automatically performed and return SNPs that are significant in the mGWAS (Figure 2c). The network output will include genes, SNPs, and metabolites. Alternatively, users can perform gene–metabolite mapping via biochemical knowledge or to the associated diseases.

### 2.4. Case Studies

#### 2.4.1. COVID-19 Case Study

**Figure 3:**
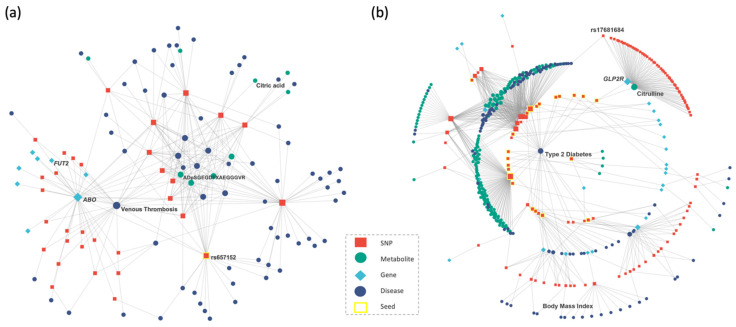
Screenshots of network results for the (**a**) COVID-19 case study and (**b**) type 2 diabetes case study. Each node represents either a SNP (orange square), a metabolite (green circle), a gene (blue diamond), a disease (dark blue circle), or a seed node (yellow square outline). Each edge is either an association between one SNP and one metabolite, an association between one SNP and one disease, a positional mapping of SNP to gene, or a protein–protein interaction. The size of a node is proportional to the number of other nodes connected to it.

The host genetic variation is known to influence the severity of SARS-CoV-2 infection [45,46,47,48,49] and the blood metabolomics can reveal biomarkers for disease diagnosis and prognosis [50,51]. However, understanding mechanisms that link genetic variation to metabolism and clinical endpoints remains an important challenge. Therefore, we applied mGWAS-Explorer to a list of SNPs identified from a GWAS of severe COVID-19 [47] to provide insights into the shared genetic architecture of diseases and intermediate metabolic phenotypes. We used a suggestive significant association *p*-value threshold (1 × 10^−5^) for mGWAS-Explorer, resulting in 19 SNPs after LD clumping. mGWAS-Explorer revealed that the SNPs at the *ABO* (alpha 1-3-N-acetylgalactosaminyltransferase and alpha 1-3-galactosyltransferase) locus were in high LD (r^2^ > 0.8) with numerous other SNPs in this region associated with multiple metabolites and other human diseases, such as leucylalanine, citric acid [51], malaria [52], ischemic stroke [53], and venous thrombosis [54]. The blood type locus *ABO* has been linked to the risk of COVID-19 in several studies [47,55]. Multiple hypotheses have been proposed to explain the mechanism, such as anti-A and/or anti-B antibodies against corresponding antigens, or the glycosyltransferase activity [56]. mGWAS-Explorer provided insights into these possible mechanisms, which identified associations of *ABO* variants with levels and the ratios of fibrinogen A-α peptides (e.g., ADpSGEGDFXAEGGGVR) and venous thromboembolism (Figure 3a). Fibrinogen plays a role in blood clotting [57]. Therefore, the association between *ABO* variants with fibrinogen may suggest that *ABO* influences COVID-19 via regulating thrombosis, which provided a functional explanation for the observed association of *ABO* with COVID-19 risk. Indeed, studies have reported that COVID-19 is associated with an increased risk of thromboembolism [58]. Therefore, we sought to investigate whether the association between fibrinogen A-α peptide-associated loci could provide additional insights into the underlying pathophysiology of COVID-19. Interestingly, mGWAS-Explorer revealed variants in *ENPEP* (glutamyl aminopeptidase) and *FUT2* (fucosyltransferase 2) genes are associated with levels and/or ratios of fibrinogen A-α peptides. Additionally, *FUT2* gene was also identified in the PPI network with the *ABO* gene (Figure 3a). In fact, *ENPEP* was discovered to be a candidate co-receptor for the coronavirus SARS-CoV-2 [59] and individuals with an inactivating *FUT2* mutations were more likely to develop a less severe form of the COVID-19 disease [60]. In summary, mGWAS-Explorer supports the evidence that *ABO*, *ENPEP*, and *FUT2* may be candidate genes and discovered fibrinogen A-α peptides as potential biomarkers for COVID-19 disease.

#### 2.4.2. Type 2 Diabetes Case Study

Around 250 genomic regions have been associated with type 2 diabetes (T2D) susceptibility in genome-wide association studies, some studies have highlighted the link to metabolomic profiles [7,61,62]. We applied mGWAS-Explorer to a list of SNPs from a published GWAS of T2D [63] in an attempt to examine shared genetic signals with circulating metabolites. Notably, mGWAS-Explorer confirmed the associations between citrulline metabolites, T2D, body mass index (Figure 3b), and identified the missense rs17681684 variant for citrulline in the *GLP2R* (glucagon like peptide 2 receptor) gene as reported by Lotta et al. [7]. Additionally, it identified shared genetic signals between T2D, coronary artery disease, and cholesterol levels at the *ABO* locus. Indeed, previous epidemiological studies have reported that the associations of *ABO* group with coronary artery diseases are mediated by cholesterol [64], although the evidence regarding associations between *ABO* blood group with type 2 diabetes were not consistent [65,66,67,68]. Thus, further studies are required to identify the associations between *ABO* variants, T2D, and cholesterol levels. Furthermore, mGWAS-Explorer also revealed metabolites levels and their ratios identified in the previous COVID-19 case study shared associations with T2D loci. In fact, multiple studies have reported the comorbidity of T2D and COVID-19 [69,70,71]. In brief, analyzing the T2D cross-phenotype associations with metabolites and other diseases highlighted comorbid conditions with shared genetic signals, illustrating the usefulness of mGWAS-Explorer.

### 2.5. Comparison with Other Tools

Table 2 provides detailed comparisons of mGWAS-Explorer with several bioinformatics resources that can be used for mGWAS, including Metabolomics GWAS Server [33,34], PheWeb [35], NETMAGE [37], and GePhEx [72]. The metabolomics GWAS server supports searching the results of two genome-wide association studies on the blood and urine metabolome in 7824 and 3861 individuals with European ancestry [33,34]. PheWeb is an excellent tool for developers to build a website to explore and visualize large-scale genetic associations [35]. NETMAGE focuses on visualizing disease–disease networks from summary statistics [37], and GePhEx allows visualization and interpretation of relationships across multiple traits with genetic associations evidence [72].

URL links:

- Metabolomics GWAS Server: http://metabolomics.helmholtz-muenchen.de/gwas/ (accessed on 1 May 2022).
- PheWeb: https://github.com/statgen/pheweb (accessed on 1 May 2022)
- NETMAGE: https://hdpm.biomedinfolab.com/netmage/ (accessed on 1 May 2022) (accept PheWAS summary statistics)
- GePhEx: https://gephex.ega-archive.org/ (accessed on 1 May 2022).

## 3. Discussion

Establishing meaningful connections between diseases and deciphering molecular mechanisms that underpin shared genetic architectures are among the key objectives of GWAS. Our work shows that integrating mGWAS summary statistics, LD proxy search, and visual analytics can rapidly reveal multiple associations across metabolites and diseases, which can be utilized to better understand the ongoing global health crisis, such as the COVID-19 pandemic and type 2 diabetes.

When looking at the cross-phenotype associations between metabolites and diseases, it is important to investigate the shared SNPs identified in the mGWAS-Explorer output to examine where the SNPs are located on the genome and the extent of overlapping of the SNPs. In fact, we consider mGWAS-Explorer as the initial stage in a pipeline for an in-depth mechanistic understanding of mGWAS before moving on to similarity analysis [26], colocalization analysis [14] or Mendelian randomization studies [73] to further investigate shared genetic signals in the same locus and to identify causal links. Ultimately, experimental studies in model organisms and human clinical studies are required to test the generated hypothesis to fully understand the mechanisms.

While our first case study highlighted shared genetic variants regulating metabolite abundance (e.g., citric acid and fibrinogen A-α peptides) and COVID-19 at *ABO*, much work needs to be done to fully understand the underlying mechanisms. Citric acid acts as a bridge between carbohydrate and fatty acid metabolism, promoting the growth and development of immune cells [74]. Additionally, citric acid is an important component in the TCA cycle. TCA cycle metabolites play key roles in signaling regulations of the innate and adaptive immune systems [75], which may be involved in COVID-19 pathogenesis. mGWAS-Explorer was also able to identify *ENPEP* and *FUT2* as potential candidate genes for COVID-19, although the association signal of these two genes were below the genome-wide significance threshold in the original study [47]. Many follow-up studies have reported *ABO* and *ENPEP* as COVID-19 risk genes; however, the evidence for the *FUT2* gene is conflicting [60,76,77]. Indeed, a recent whole-genome sequencing study identified variants in *FUT2* associated with critical COVID-19 diseases [45]. *FUT2* is responsible for the expression of histo-blood group antigens on the mucosal surface of gastrointestinal, genitourinary, and respiratory tracts. Inactivating *FUT2* mutations lead to a non-secretor status, which confer resistance to norovirus and rotavirus gastrointestinal infections [78,79]. Furthermore, the minor allele frequency of the stop–gain variant rs601338 in the *FUT2* gene is drastically different between the European population (0.441) and the East Asian population (0.004) [80,81], which might explain the differences in host response to SARS-CoV-2 among different populations. However, the mechanism by which secretor status influencing COVID-19 pathogenesis is not fully understood. Therefore, it may be valuable to perform colocalization analysis and Mendelian randomization studies to identify the causal link between *FUT2* variants, fibrinogen A-α peptides, and COVID-19.

Increases in throughput and decreases in cost will enable a growing number of mGWAS to be conducted in the near future. The web server will be regularly updated to incorporate the most up-to-date mGWAS datasets, disease associations, and additional SNP annotation data (e.g., eQTLs or chromatin interactions) to serve as a valuable bioinformatics platform for mGWAS researchers. With this context, we also intend to add support for peak annotations of untargeted metabolomics data obtained from high-resolution mass spectrometry.

## 4. Materials and Methods

### 4.1. Knowledgebase Curation

(a) mGWAS papers were searched from PubMed, Web of Science, bioRxiv, and medRxiv, resulting in 65 publications as of December 2021. The summary statistics were either downloaded from public databases or supplementary data of the original publications. Statistical associations between metabolites and SNPs were summarized and pre-filtered using study-specific significance thresholds. In addition to *p*-values and effect sizes of SNP–metabolite associations, we have included metadata from each publication, such as the type of biofluid, sample size, population type, genotyping platform, metabolomics platform, etc. (b) For SNP annotation, three options are provided, including HaploReg [82], PhenoScanner [28], and VEP [20] by using the Application Programming Interface (API) service of each database. For the first two options, users can also perform an LD proxy search based on different populations and r^2^ values. With VEP, users can select either a specific distance or the nearest number of genes for SNP annotation. (c) SNP–disease and gene–disease associations were downloaded from the DisGeNET database [32]. HMDB database was used to obtain metabolite–disease associations [83]. (d) KEGG, Recon3D, and Transporter Classification Database (TCDB) were used to curate knowledge-based gene–metabolite association information [84,85,86]. (e) The protein–protein interaction information is based on several well-established PPI databases [87,88,89]. (f) The libraries for enrichment analysis were curated from seven well-known databases, including GO, Reactome, KEGG, Orphanet, DrugMatrix, DisGeNET and DSigDB. The detailed description of these databases and their links can be found in the Supplementary Material.

### 4.2. Input Processing and Connection Identification

SNPs are identified by rsIDs, genes are identified by Entrez IDs, and metabolites are identified by HMDB IDs, platform-specific IDs, or feature tags (m/z_retention_time). Additional identifiers have also been included, such as genomic coordinates for SNPs (after lifting all SNPs to GRCh37 assembly using LiftOver [90]), Ensemble ID, and gene symbol for genes, as well as KEGG ID and common name for metabolites.

There are two general types of relationships, including inter-omics and phenotype-specific links. Inter-omics connections are based on statistical associations (based on mGWAS), or knowledge-based associations (based on positional mapping for SNP–gene annotation or encoding enzymes/transporters for metabolite–gene connections). Phenotype-specific links allow users to identify variants that are associated with disease phenotypes. This information is obtained from DisGeNET [32] based on case-control genome-wide association studies or via text mining from the literature.

### 4.3. Implementation

The backend analysis was implemented using the R programming language (version 4.1.3). The whole framework was built based on the PrimeFaces component library (version 11.0.0). The integrated data are stored in a relational database using SQLite. The interactive visualization was developed based on the sigma.js and echarts.js JavaScript libraries for network view and 3D Manhattan plot, respectively.

### 4.4. Data Collection for Case Studies

The datasets of COVID-19 and type 2 diabetes case studies were downloaded from their respective original publications [47,63]. LD clumping were performed to identify the independent signals by using the *ieugwasr* package with default parameters [29] prior to the analysis. Specifically, the SNPs with the lowest *p*-value are retained, where SNPs in LD within a certain window are removed in LD clumping [91]. In both case studies, European population and r^2^ = 0.8 were set as input parameters for LD proxy search.

### 4.5. Network Visual Analytics

#### 4.5.1. Network Creation and Customization

The default networks are built by querying for the direct mapping from the knowledgebase. Optionally, users can choose to expand the network by including PPIs in the SNP module. However, the result may suffer from the ‘hairball’ effect, which severely limits the usefulness and interpretability. Therefore, mGWAS-Explorer offers support to refine large networks based on node degree or betweenness values, batch filtering, or the shortest paths, as well as by computing minimum subnetworks based on the prize-collecting Steiner Forest (PCSF) algorithm [92]. The detailed instructions on how to navigate the network visualization system can be found in our Supplementary Material.

#### 4.5.2. Functional Enrichment Analysis

The combination of network visualization and functional enrichment analysis is a valuable tool for gaining key biological insights. For SNP input, two types of enrichment approaches have been implemented—(1) directly testing in SNP-set library, or (2) testing on mapped genes for enrichment using hypergeometric tests. When the input is a gene or metabolite, the associated gene-set or metabolite-set enrichment analysis can be performed. The result tables will be displayed under the Function Explorer panel. Notably, clicking a row of the table will highlight the nodes contained in the corresponding function/pathway within the network. In addition, mGWAS-Explorer also permits enrichment analysis on the selected nodes of interests, for instance, from the batch selection panel.

#### 4.5.3. Other Advanced Features

The Network Viewer page contains multiple advanced features for network visual exploration, including Network Layout, Global Node Styles, Module Explorer, Batch Selection, and Path Finder. Ten different network layout algorithms are available, including Force-Atlas, Fruchterman–Reingold, Circular, Graphopt, Large Graph, Random, Circular Bipartite/Tripartite, Linear Bipartite/Tripartite, Concentric, and Backbone layout. Three module detection algorithms are offered in the Module Explorer, including the WalkTrap, InfoMap, and the Label Propagation algorithms based on the igraph R package [93]. These options can be combined to obtain a better visualization experience. Users can find details of these algorithms on our FAQs page.

## 5. Conclusions

We have developed mGWAS-Explorer to allow users to easily explore the published mGWAS datasets, and to provide contextualized analysis for a given list of SNPs, genes, or metabolites. As demonstrated by our case studies of COVID-19 and type 2 diabetes, mGWAS-Explorer can facilitate hypothesis generation and reveal functional insights into the genetic basis of human metabolism to permit translational discoveries.

## Figures and Tables

**Table 1:** A summary of the mGWAS datasets in mGWAS-Explorer.

Sample Type	Study #	* Metabolite #	** Metabolite Ratio #	SNP #	SNP–Metabolite Associations #
Blood	57	3992	1265	67,570	30,3090
Urine	5	271	1123	6877	9647
Saliva	1	14	0	1364	1454
Cerebrospinal fluid (CSF)	1	15	0	1178	1182
Mitochondria	1	0	390	194	404
Sum (unique)	65	4147	2388	73,737	313,720

* Metabolite number includes both targeted (compound names) and untargeted measures (feature IDs, such as ‘391.2859_3.774′ based on mass to charge ratio and retention time. The total number of such feature IDs is 2464). ** Metabolite ratios (metabolite A/metabolite B) can be useful as they may reflect the biochemical conversion of metabolites and thus enhance the association signals. The # sign indicates size or total number [43].

**Table 2:** Comparison of the main features of mGWAS-Explorer with other web-based tools. Symbols used for feature evaluations with ‘√’ for present, ‘−’ for absent, and ‘+’ for a more quantitative assessment (more ‘+’ symbols indicate better support).

Tool Name	mGWAS-Explorer	Metabolomics GWAS Server	PheWeb	NETMAGE	GePhEx
**Data input and processing**					
SNP	√	√	√	√	√
LD proxy search	√	√	−	−	√
Gene	√	√	√	−	√
Metabolite	√	√	√	−	√
**Enrichment analysis**					
SNP-set	√	−	−	−	−
Gene-set	√	−	−	−	√
Metabolite-set	√	−	−	−	−
**Cross-phenotype exploration**	√	√	√	√	√
**Visual analytics**					
Network visualization	+++	−	−	+	−
Network customization	+++	−	−	+	−
Integration with PPI network	√	−	−	−	−
Subnetwork extraction	√	−	−	√	−
Topology-based filtering	√	−	−	−	−
3D Manhattan plot	√	−	−	−	−

## Data Availability

The data is available from https://www.mgwas.ca/mGWAS/faces/Secure/Resources.xhtml (accessed on 1 May 2022).

## Supplementary Materials

The following supporting information can be downloaded at: https://www.mdpi.com/article/10.3390/metabo12060526/s1, File S1: mGWAS-Explorer Program Description and Methods [20,28,32,82,84,85,86,87,88,89,94,95,96,97,98,99].
