# Supplementary material for: mGWAS-Explorer: Linking SNPs, Genes, Metabolites, and Diseases for Functional Insights
Source: Metabolites. 2022 Jun 7;12(6):526. doi: 10.3390/metabo12060526 (PMC9230867; doi:10.3390/metabo12060526)
Supplement: Supplementary file 1 [file metabolites-12-00526-s001.zip › metabolites-1730261-supplementary.pdf]

Supplementary material to

mGWAS-Explorer: linking SNPs, genes, metabolites and diseases for functional insights

PROGRAM DESCRIPTION AND METHODS

1. KNOWLEDGEBASE CREATION 2

1.1 Knowledgebase for network creation 2

1.1.1 SNP-metabolite association (mGWAS) 2

1.1.2 SNP to gene mapping 2

1.1.3 LD proxy search 2

1.1.4 SNP-disease association 2

1.1.5 Gene-metabolite association 3

1.1.6 Protein-protein interaction 3

1.1.7 Gene-disease association 3

1.1.8 Metabolite-disease association 3

1.2 Knowledgebase for network interpretation 3

1.2.1 SNP-sets 4

1.2.2 Gene-sets 4

1.2.3 Metabolite-sets 4

2. DATA SEARCH 4

3. DATA EXPLORE 4

4. DATA UPLOAD AND PROCESSING 5

4.1 Data inputs 5

5. NETWORK CREATION AND REFINEMENT 5

6. NETWORK VISUALIZATION AND FUNCTIONAL ANALYSIS 6

6.1 General network customization 7

6.2 Node searching and edge viewing 7

6.3 Functional enrichment analysis 8

6.4 Other Advanced Features 8

7. IMPLEMENTATION 8

8. REFERENCES 9

1. KNOWLEDGEBASE CREATION

1.1 Knowledgebase for network creation

1.1.1 SNP-metabolite association (mGWAS)

As of December 2021, 65 mGWAS papers had been found after searching PubMed, Web of Science, bioRxiv, and medRxiv, after a thorough literature research. The summary statistics were either collected from publicly available databases or supplementary data from the original publications. Study-specific significant thresholds were used to pre-filter statistical associations between metabolites and SNPs. Details of the curated mGWAS dataset can be found in our Resources page: https://www.mgwas.ca/mGWAS/faces/Secure/Resources.xhtml

1.1.2 SNP to gene mapping

Three options are provided for SNP to gene mapping, including HaploReg [1], PhenoScanner [2], and VEP [3] by using the Application Programming Interface (API) service of each database.

The Ensembl Variant Effect Predictor (VEP) is a comprehensive suite of tools for analyzing, annotating, and prioritizing genomic variants in both coding and non-coding regions.

https://rest.ensembl.org/documentation/info/vep_id_get

PhenoScanner is a curated database of results from large-scale genetic association studies.

https://github.com/phenoscanner/phenoscanner

HaploReg is a database for mining putative causal variants, cell types, regulators, and target genes for human diseases and complex traits.

https://github.com/izhbannikov/haploR

1.1.3 LD proxy search

mGWAS-Explorer allow users to search for metabolites/diseases associations with proxies for SNPs of interest using the HaploReg API or PhenoScanner API. The LD information is based on the 1000 Genomes Project.

1.1.4 SNP-disease association

DisGeNET was used to obtain SNP-disease associations, which contains both curated and literature data [4]. The curated data include SNP-disease associations from UniProt [5], ClinVar [6], GWAS Catalog [7], and GWASdb [8].

https://www.disgenet.org/downloads

1.1.5 Gene-metabolite association

Knowledge-based gene-metabolite association information was curated using KEGG, Recon3D, and the Transporter Classification Database (TCDB) [9-11].

KEGG: metabolite-gene associations based on KEGG reaction.

https://www.genome.jp/kegg/

Recon3D: high-quality genome-scale metabolic reconstruction.

https://www.vmh.life/

TCDB: transporter classification database for transporter protein information.

https://www.tcdb.org/

1.1.6 Protein-protein interaction

Information on protein-protein interaction was taken from four well-established PPI databases, including InnateDB [12], STRING [13], HuRI [14], and Rolland et al [15].

InnateDB contains literature-curated data on protein-protein interactions.

https://www.innatedb.com/index.jsp

STRING is a comprehensive database for protein-protein interaction networks. We have filtered on medium (400) to high (900) confidence score.

https://string-db.org/

HuRI is a reference interactome map of human binary protein interactions.

http://www.interactome-atlas.org/

Rolland et al. contains experimentally validated binary human PPI data.

http://interactome.dfci.harvard.edu/H_sapiens/

1.1.7 Gene-disease association

DisGeNET was also used to obtain gene-disease associations.

https://www.disgenet.org/downloads

1.1.8 Metabolite-disease association

HMDB was used to retrieve metabolite-disease associations.

https://hmdb.ca/downloads

1.2 Knowledgebase for network interpretation

For network analysis, it is crucial to be able to interpret the results in addition to visualization. In this regard, enrichment analysis plays a key role. Thus, we have implemented three types of enrichment analysis, including SNP-set, gene-set and metabolite-set enrichment analysis.

1.2.1 SNP-sets

DisGeNET was used for SNP-sets, where diseases having three or more SNPs are taken into account when creating a SNP-set.

https://www.disgenet.org/downloads

1.2.2 Gene-sets

Gene Ontology (GO) was used to obtain gene-sets for biological processes, molecular functions, and cellular components.

http://geneontology.org/

Reactome and KEGG was used to get gene-sets for pathways.

https://reactome.org/download-data

https://www.kegg.jp/

Orphanet was used to obtain gene-sets for rare diseases.

http://www.orphadata.org/cgi-bin/index.php

DrugMatrix and DSigDB were used to obtain gene-sets that related drugs and their target genes.

https://ntp.niehs.nih.gov/data/drugmatrix/

http://dsigdb.tanlab.org/DSigDBv1.0/

1.2.3 Metabolite-sets

KEGG was used to retrieve metabolite-sets for KEGG pathways.

https://www.kegg.jp/

2. DATA SEARCH

The 'Search' module allows users to search the results for significant SNP-metabolite associations from the curated mGWAS datasets. Users can enter the rsID or Common Name in the search bar, where autocomplete is supported. The search results will return in the table below.

3. DATA BROWSE

The 'Browse' module allows users to visually examine the summary statistics from individual mGWAS datasets in a 3D Manhattan plot. Users can zoom in/out or rotate the 3D plot and mouse over on the dots to see detailed information. Meanwhile, table view and network view are also provided.

4. DATA UPLOAD AND PROCESSING

4.1 Data inputs

The flexible interface allows users to start from SNPs, genes, or metabolites. The input can be uploaded by entering a list of IDs (SNPs, genes, metabolites). Users can refer to the relevant FAQs and tutorials or see our test examples for more details.

mGWAS-Explorer currently supports rsID for SNPs, Ensembl gene ID, Entrez ID and official gene symbol for genes, HMDB ID, KEGG ID and compound name for metabolites. Additionally, users can filter on “Biofluid” or “Population”, the results will return SNP-metabolite associations on the metabolites measured in the chosen biofluid and population.

5. NETWORK CREATION AND REFINEMENT

The input data from users will be searched against our knowledgebase. The resulting pair-wise tables are used to generate the default networks. Since not all nodes will be connected, the results may return many networks, usually one large network with a few smaller ones. Table summary and network summary are displayed (Figure S1), indicating the statistics for nodes and edges to allow users to have an overview of the networks. We recommend that users keep their networks below 2000 nodes for practical reasons, since large networks will induce a ‘hairball’ effect, which make it difficult to comprehend the results. mGWAS-Explorer has built-in network tools that allow users to refine networks according to topological measurements (degree, betweenness, and shortest path), batch filtering, and computing minimum subnetworks based on the prize-collecting Steiner Forest (PCSF) algorithm.

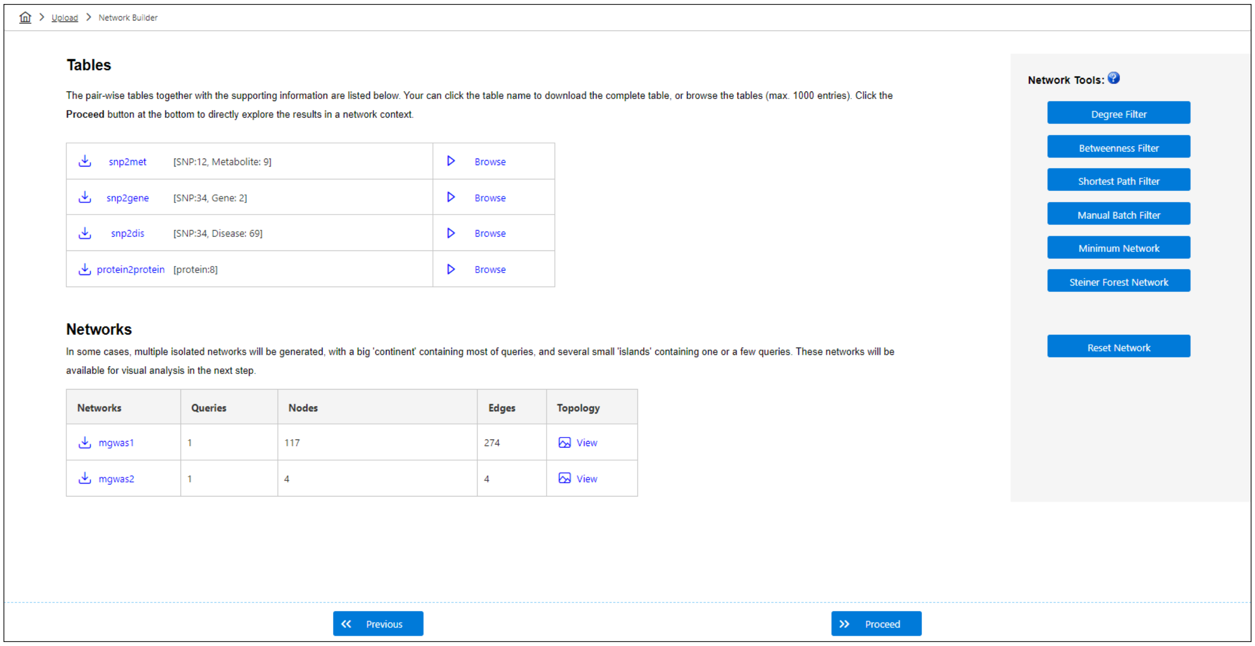

Figure S1. A screenshot of the Network Builder page, including table statistics, network statistics and network tools.

6. NETWORK VISUALIZATION AND FUNCTIONAL ANALYSIS

The HTML5 canvas and JavaScript were used to develop the network visualization system. Figure S2 shows a screenshot of the network visualization interface. The network visualization system comprises four main components: the top menu bar, the left node table, the center network viewing area, and the right panel.

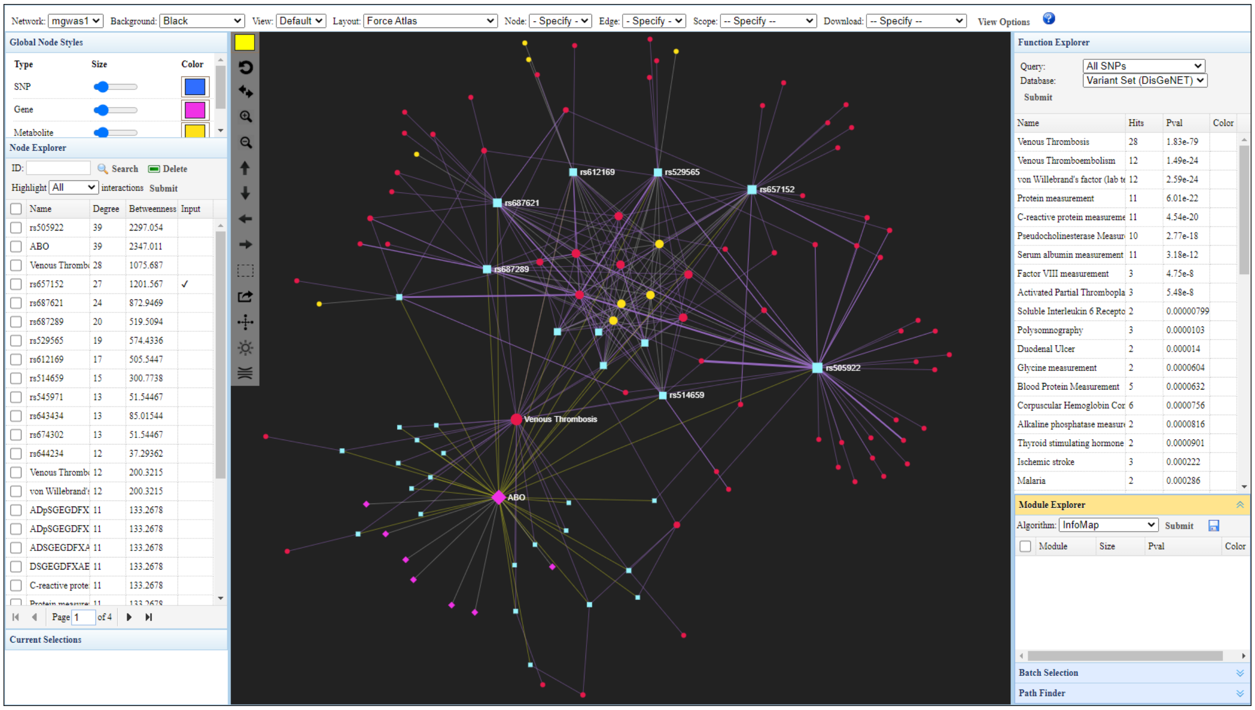

Figure S2. A screenshot of the mGWAS-Explorer network visual analytic system. The system comprises four main components: the top menu bar, the left node table, the center network viewing area, and the right panel. Users can easily highlight and arrange nodes based on their connectivity patterns or enriched functions.

6.1 General network customization

The top menu bar offers common functions to customize the network, such as changing the background color, adjusting the characteristics of the nodes (label, color, size, shape) and edges (opacity, thickness, color), or downloading the results. Users can select the preferred network layout by using the 'Layout' option. The 'Scope' option allows users to specify the mouse operation range during drag-and-drop, either 'Single node', 'Node-neighbors', 'All highlights', 'Current highlights', or 'Node type'.

6.2 Node searching and edge viewing

The nodes of the network are displayed in the Node Explorer on the left side, along with their degree and betweenness measurements. A checkmark will show in the 'Input' column if it is a seed node provided by the user. Users can click on a row or type the ID in the search bar to view the node of interest. The network will zoom to the selected node automatically. Alternatively,

multiple nodes can be selected by clicking the checkboxes. Users can decide to highlight 'All' or the 'Shared' nodes accordingly. Meanwhile, double clicking an edge will show the evidence that supports the connection between the nodes.

6.3 Functional enrichment analysis

The combination of network visualization and functional enrichment analysis can provide valuable biological insights. mGWAS-Explorer supports over representation analysis (ORA) [16]. ORA is a widely used method to assess whether known biological functions or pathways are over-represented (i.e., enriched) in a list of interest (e.g., SNP, gene, or metabolite). Hypergeometric tests are used to calculate the p-values.

6.4 Other Advanced Features

The bottom right panel contains three tabs – *Module Explorer*, *Batch Selection*, and *Path Finder*. The *Module Explorer* tab provides three different approaches for module detection – the WalkTrap, InfoMap, and Label propagation algorithms. Users can perform module detection to identify tightly clustered subnetworks with more internal connections than would be expected at random in the whole network. The *Batch Selection* tab allows users to highlight or exclude a list of nodes. The *Path Finder* tab allows users to find the shortest path between any two nodes.

7. IMPLEMENTATION

The backend of mGWAS-Explorer was implemented using the R programming language (version 4.1.3; https://www.r-project.org/). The whole framework was built based on the JavaServer Faces technology using the PrimeFaces component library (version 11.0; https://www.primefaces.org/). The integrated data is stored in a relational database using SQLite (https://www.sqlite.org/index.html). Network visualization and analysis are based on *jquery* (https://jquery.com/) for general-purpose scripting, *sigma.js* (https://www.sigmajs.org/) for network display and interactions, and *igraph* (https://igraph.org/) for network analysis and layout. 3D Manhattan plot is built on *ECharts-GL*, an extension pack of Apache ECharts, which provides 3D plots and WebGL acceleration (https://echarts.apache.org/en/index.html).

8. REFERENCES

1. Ward, L.D.; Kellis, M. HaploReg v4: systematic mining of putative causal variants, cell types, regulators and target genes for human complex traits and disease. *Nucleic acids research* **2015**, *44*, D877-D881.
2. Kamat, M.A.; Blackshaw, J.A.; Young, R.; Surendran, P.; Burgess, S.; Danesh, J.; Butterworth, A.S.; Staley, J.R. PhenoScanner V2: an expanded tool for searching human genotype-phenotype associations. *Bioinformatics* **2019**, *35*, 4851-4853, doi:10.1093/bioinformatics/btz469.
3. McLaren, W.; Gil, L.; Hunt, S.E.; Riat, H.S.; Ritchie, G.R.S.; Thormann, A.; Flicek, P.; Cunningham, F. The Ensembl Variant Effect Predictor. *Genome Biology* **2016**, *17*, 122, doi:10.1186/s13059-016-0974-4.
4. Piñero, J.; Ramírez-Anguita, J.M.; Saüch-Pitarch, J.; Ronzano, F.; Centeno, E.; Sanz, F.; Furlong, L.I. The DisGeNET knowledge platform for disease genomics: 2019 update. *Nucleic Acids Res* **2020**, *48*, D845-d855, doi:10.1093/nar/gkz1021.
5. UniProt Consortium, T. UniProt: the universal protein knowledgebase. *Nucleic acids research* **2018**, *46*, 2699, doi:10.1093/nar/gky092.
6. Landrum, M.J.; Kattman, B.L. ClinVar at five years: Delivering on the promise. *Human mutation* **2018**, *39*, 1623-1630, doi:10.1002/humu.23641.
7. MacArthur, J.; Bowler, E.; Cerezo, M.; Gil, L.; Hall, P.; Hastings, E.; Junkins, H.; McMahon, A.; Milano, A.; Morales, J.; et al. The new NHGRI-EBI Catalog of published genome-wide association studies (GWAS Catalog). *Nucleic acids research* **2017**, *45*, D896-d901, doi:10.1093/nar/gkw1133.
8. Li, M.J.; Liu, Z.; Wang, P.; Wong, M.P.; Nelson, M.R.; Kocher, J.P.; Yeager, M.; Sham, P.C.; Chanock, S.J.; Xia, Z.; et al. GWASdb v2: an update database for human genetic variants identified by genome-wide association studies. *Nucleic acids research* **2016**, *44*, D869-876, doi:10.1093/nar/gkv1317.
9. Kanehisa, M. Enzyme annotation and metabolic reconstruction using KEGG. *Protein Function Prediction: Methods and Protocols* **2017**, 135-145.
10. Saier, M.H.; Reddy, V.S.; Moreno-Hagelsieb, G.; Hendargo, K.J.; Zhang, Y.; Iddamsetty, V.; Lam, K.J.K.; Tian, N.; Russum, S.; Wang, J.; et al. The Transporter Classification Database (TCDB): 2021 update. *Nucleic Acids Res* **2021**, *49*, D461-d467, doi:10.1093/nar/gkaa1004.

11. Brunk, E.; Sahoo, S.; Zielinski, D.C.; Altunkaya, A.; Dräger, A.; Mih, N.; Gatto, F.; Nilsson, A.; Preciat Gonzalez, G.A.; Aurich, M.K.; et al. Recon3D enables a three-dimensional view of gene variation in human metabolism. *Nature biotechnology* **2018**, *36*, 272-281, doi:10.1038/nbt.4072.

12. Breuer, K.; Foroushani, A.K.; Laird, M.R.; Chen, C.; Sribnaia, A.; Lo, R.; Winsor, G.L.; Hancock, R.E.; Brinkman, F.S.; Lynn, D.J.J.N.a.r. InnateDB: systems biology of innate immunity and beyond—recent updates and continuing curation. **2013**, *41*, D1228-D1233.

13. Szklarczyk, D.; Gable, A.L.; Lyon, D.; Junge, A.; Wyder, S.; Huerta-Cepas, J.; Simonovic, M.; Doncheva, N.T.; Morris, J.H.; Bork, P.; et al. STRING v11: protein-protein association networks with increased coverage, supporting functional discovery in genome-wide experimental datasets. *Nucleic acids research* **2019**, *47*, D607-d613, doi:10.1093/nar/gky1131.

14. Luck, K.; Kim, D.K.; Lambourne, L.; Spirohn, K.; Begg, B.E.; Bian, W.; Brignall, R.; Cafarelli, T.; Campos-Laborie, F.J.; Charloteaux, B.; et al. A reference map of the human binary protein interactome. *Nature* **2020**, *580*, 402-408, doi:10.1038/s41586-020-2188-x.

15. Rolland, T.; Taşan, M.; Charloteaux, B.; Pevzner, S.J.; Zhong, Q.; Sahni, N.; Yi, S.; Lemmens, I.; Fontanillo, C.; Mosca, R.J.C. A proteome-scale map of the human interactome network. **2014**, *159*, 1212-1226.

16. Boyle, E.I.; Weng, S.; Gollub, J.; Jin, H.; Botstein, D.; Cherry, J.M.; Sherlock, G. GO::TermFinder--open source software for accessing Gene Ontology information and finding significantly enriched Gene Ontology terms associated with a list of genes. *Bioinformatics (Oxford, England)* **2004**, *20*, 3710-3715, doi:10.1093/bioinformatics/bth456.
